# Urethral Stricture Associated With an Artificial Urethral Sphincter: A Case Report

**DOI:** 10.1002/iju5.70049

**Published:** 2025-05-23

**Authors:** Akira Tachibana, Kazumasa Torimoto, Daisuke Gotoh, Kenta Onishi, Shunta Hori, Yosuke Morizawa, Yasushi Nakai, Makito Miyake, Nobumichi Tanaka, Kiyohide Fujimoto

**Affiliations:** ^1^ Department of Urology Nara Medical University Kashihara Nara Japan; ^2^ Department of Urology Nara Prefecture General Medical Center Nara Nara Japan; ^3^ Department of Prostate Brachytherapy Nara Medical University Kashihara Nara Japan

**Keywords:** artificial urinary sphincter, Heineke–Mikulicz principle, non‐transecting, urethral stricture, urethroplasty

## Abstract

**Introduction:**

Artificial urinary sphincter implantation is the standard treatment for moderate‐to‐severe stress urinary incontinence in men. We report a case of urethral stricture associated with an artificial urinary sphincter in a patient who underwent urethroplasty and subsequent replacement of the implant.

**Case Presentation:**

A 64‐year‐old man who had undergone radical retropubic prostatectomy presented to our department for stress urinary incontinence. An artificial urinary sphincter was implanted, and the incontinence resolved. Eleven years later, the patient returned because of voiding symptoms. Urethroscopy and retrograde urethrography revealed a pendulous urethral stricture, measuring approximately 2 mm in length. The patient underwent implant removal and non‐transecting anastomotic urethroplasty. Postoperative urethroscopy confirmed resolution of the stricture. Artificial urinary sphincter reimplantation was performed 6 months later, restoring continence.

**Conclusion:**

Non‐transecting urethroplasty to treat short urethral strictures associated with an artificial urinary sphincter may increase the success of artificial sphincter replacement.

**Summary**

- We describe a case of urethral stricture not associated with urethral erosion following artificial urinary sphincter implantation.
- We selected non‐transecting urethroplasty as the surgical technique to protect the retrograde blood supply.
- The urethra at the stricture was incised longitudinally and sutured transversely.
- The non‐transecting anastomotic urethroplasty was successful and allowed for subsequent artificial urinary sphincter reimplantation.

**Abbreviations**
AUS: artificial urinary sphincter
EPA: excision and primary anastomosis
RRP: radical retropubic prostatectomy
SUI: stress urinary incontinence

## 1 Introduction

Artificial urinary sphincter (AUS) implantation is the standard treatment for males with moderate‐to‐severe stress urinary incontinence (SUI) [1]. A retrospective study quantified the potential complications after AUS placement, including urinary retention in 31% of patients, device infection in 2%, and urethral erosion in 2% [2]. The presence of urethral erosion can lead to the development of urethral stricture or device infection [3]. However, the rate of urethral stricture not associated with erosion after AUS placement is unknown.

Herein, we describe a patient with a urethral stricture that developed at the site of AUS cuff placement without erosion, who was treated with urethroplasty and subsequent implant replacement.

## 2 Case Presentation

**FIGURE 1:**
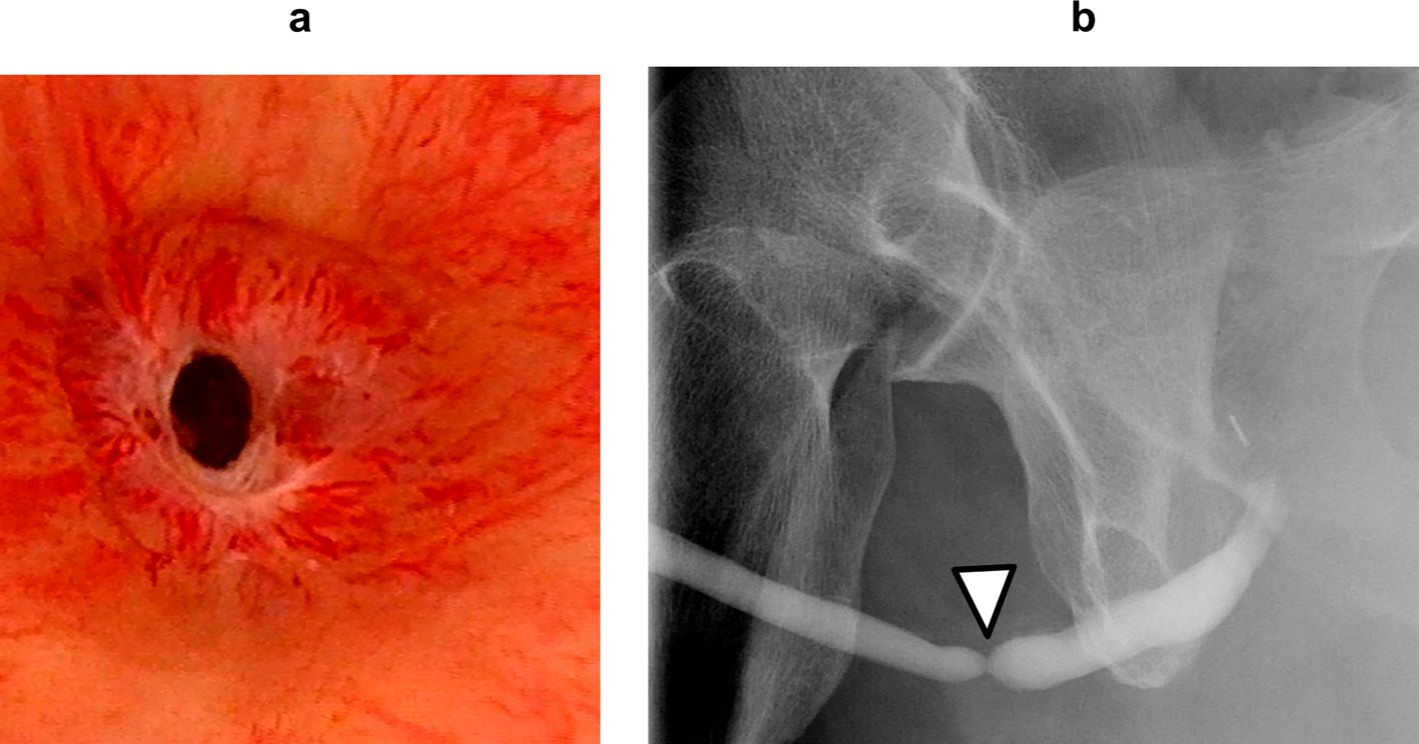
(a) Cystoscopy showing the bulbar urethral stricture. (b) Retrograde urethrography showing the length of the stricture of approximate 2 mm (white arrow).

**FIGURE 2:**
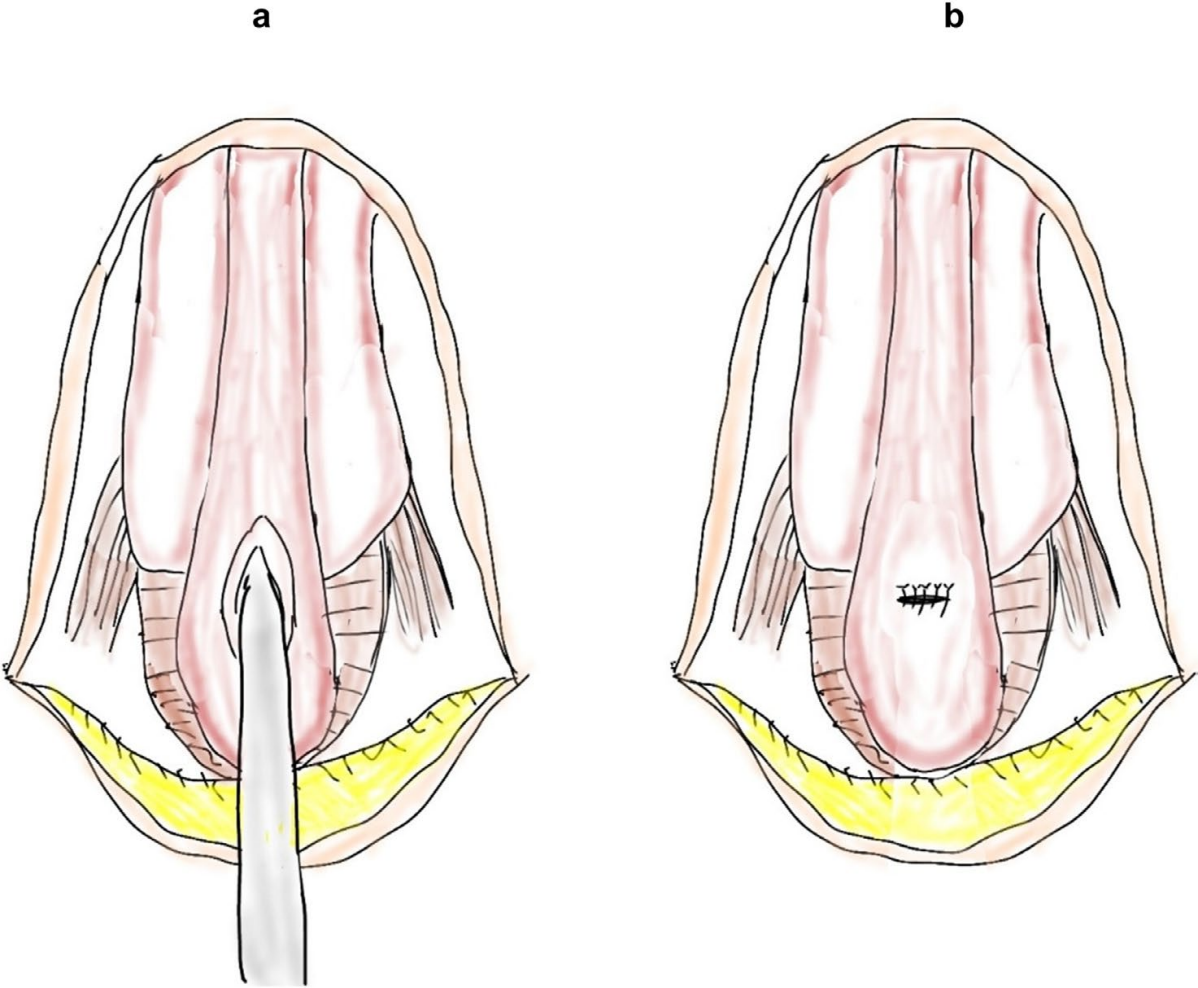
(a) The bulbar urethral, with longitudinal incision. (b) Placement of transversal sutures (Heineke–Mikulicz principle).

**FIGURE 3:**
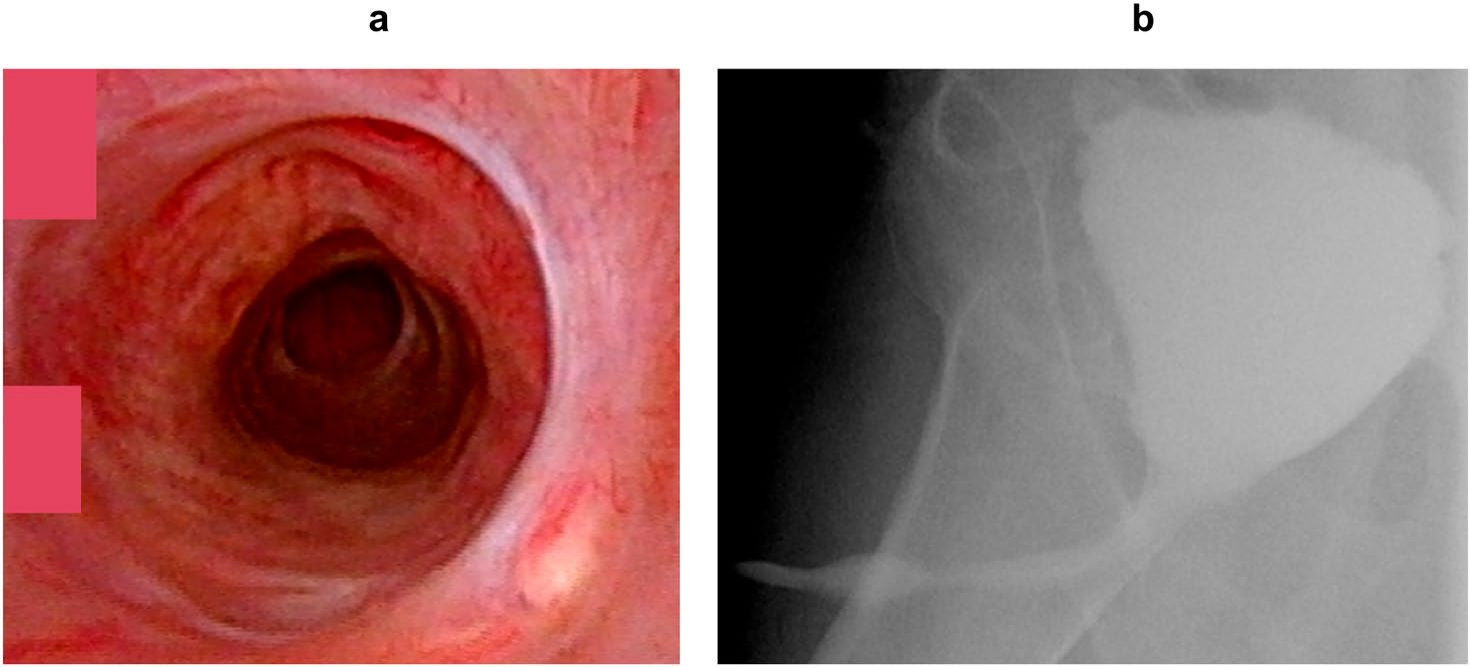
(a) Postoperative cystoscopy showing reversal of the urethral stricture. (b) Voiding cystourethrography revealing the normal urinary stream.

A 64‐year‐old man with SUI was referred to our department. He had undergone radical retropubic prostatectomy (RRP) for prostate cancer 2 years previously. He lost sexual function including erectile function after the RRP. Urethroscopy showed no urethral stricture, and an AUS (AMS800; Boston Scientific Corporation) with a cuff size of 4.5 cm was implanted. The AUS was activated after 2 months, and urinary continence was restored. Eleven years after AUS implantation, the patient returned to our department with voiding symptoms. Urethroscopy revealed a pendulous urethral stricture (Figure 1a). There was no evidence of cuff erosion. The stricture site coincided with the location of the AUS cuff. Retrograde urethrography revealed a stricture measuring approximately 2 mm in length (Figure 1b). The patient underwent removal of the AUS, urethroplasty, and percutaneous cystostomy. Normally, the urethra receives both antegrade and retrograde blood flow from the dorsal penile artery. However, in the present case, the proximal urethra had already been transected because of the RRP. We selected non‐transecting urethroplasty as the surgical technique to protect the retrograde blood supply. The urethra at the stricture was incised longitudinally and sutured transversely (Heineke–Mikulicz principle [4]) (Figure 2a,b). Postoperative urethroscopy and voiding cystourethrography revealed resolution of the urethral stricture (Figure 3a,b). After the procedures, the patient needed to use 5–6 pads for 24 h due to urinary incontinence. The patient underwent transcorporeal AUS replacement 6 months after the urethroplasty. A post‐urethroplasty urethroscopy showed no scarring in the urethra. Thus, we determined that the urethral blood flow was sufficient and reimplanted a 4.5 cm AUS cuff in the same location. The AUS device was activated 2 months after replacement. Nine months after the replacement, the patient was continent and had not experienced any complications.

## 3 Discussion

Transurethral treatments are recommended for urethral strictures in cases that meet the following criteria: not related to trauma; no history of previous treatment; strictures less than 2 cm in length; solitary strictures; and limited to the bulbar urethra [5]. In all other cases, urethroplasty is recommended [5]. The efficacy of transurethral treatment for penile and distal bulbar urethral strictures was low in a prospective randomized study [6]. We selected urethroplasty in the present case because the urethral stricture site was pendulous. Furthermore, considering the risk of exposure of the AUS cuff and device‐related infections, transurethral treatment was deemed inappropriate. The European Association of Urology guidelines do not recommend the use of anastomotic urethroplasty for penile urethral strictures due to the risk of postoperative chordee [7]. Anastomotic urethroplasty can be offered in selected cases of short (< 1 cm) penile strictures related to trauma [7]. Shakir et al. reported a success rate of 93% in 14 patients who underwent excision and primary anastomosis (EPA) for traumatic strictures of the pendulous urethra, without erectile dysfunction or penile curvature [8]. The corpus spongiosum penis is supplied by two blood flows: antegrade from the bulbar artery and retrograde from the dorsal penile artery through the corpus cavernous penis and glans [5]. Since the corpus spongiosum penis is circumferentially incised in EPA, the antegrade blood flow is interrupted, which may lead to sexual dysfunction. When performing urethroplasty, urethral transection should be avoided in cases with hypospadias, a history of radiation therapy or RPP, or when there is a possibility of AUS implantation [9]. In the present case, we selected non‐transecting urethroplasty because the patient had undergone RPP, was under consideration for a repeat AUS implantation, and the stricture length was short. Although the direct cause of the stricture was unclear, strictures can be caused by ischemia, inflammation, or urethral atrophy due to compression of the AUS cuff. The Heineke–Mikulicz principle is a technique for treating strictures by longitudinal incision and transverse closure [4]. Lumen et al. reported a success rate of 90% using the Heineke–Mikulicz principle in 10 patients with urethral strictures [4]. We were able to repair the stricture associated with AUS by preforming the Heineke–Mikulicz technique to preserve urethral blood flow without disrupting the corpus spongiosum penis. Adamakis et al. [10] reported that holmium: yttrium‐aluminum‐garnet laser ablation and endoscopic incision of the stricture using a pediatric resectoscope were effective for the treatment of recurrent strictures at the vesicourethral anastomosis after RPP and AUS implantation. However, there have been few reports of treatment for urethral strictures associated with AUS implantation, and no established treatment has been reported. Urethroplasty without cutting the corpus spongiosum penis is an option and may allow for the subsequent replacement of AUS. Further follow‐up is required to evaluate for stricture recurrence.

In conclusion, we describe a case of urethral stricture associated with AUS implantation. Non‐transecting urethroplasty is a surgical technique that shows promise as an effective treatment to make subsequent replacement of an AUS possible.

## Consent

We obtained consent from the patient for publication.

## Conflicts of Interest

The authors declare no conflicts of interest.
